# Diverse Expression Patterns of Subgroups of the *rif* Multigene Family during *Plasmodium falciparum* Gametocytogenesis

**DOI:** 10.1371/journal.pone.0003779

**Published:** 2008-11-20

**Authors:** Michaela Petter, Insa Bonow, Mo-Quen Klinkert

**Affiliations:** Bernhard Nocht-Institute for Tropical Medicine, Hamburg, Germany; Walter and Eliza Hall Institute of Medical Research, Australia

## Abstract

**Background:**

The maturation of *Plasmodium falciparum* gametocytes in the human host takes several days, during which the parasites need to efficiently evade the host immune system. Like asexual stage parasites, immature gametocytes can sequester at various sites in the human body, and only mature sexual stages are found in the circulation. Although the fundamental mechanisms of gametocyte immune evasion are still largely unknown, candidate molecules that may be involved include variant antigens encoded by multigene families in the *P. falciparum* genome, such as the PfEMP1, STEVOR and RIFIN proteins. While expression of the former two families in sexual stages has been investigated earlier, we report here RIFIN expression during gametocytogenesis.

**Methodology/Principal Findings:**

Variants of two previously characterized RIFIN subfamilies (A- and B-type RIFINs) were found to be synthesized in gametocytes. Immunofluorescence experiments showed A-type RIFINs to be accumulated in a crescent-shaped pattern of discrete punctate structures at the infected erythrocyte membrane, while members of the B-type family were associated with the parasite. Transcription analysis demonstrated the existence of diverse transcriptional regulation patterns during sexual differentiation and indicated variant-specific regulation of B-type RIFINs, in contrast to group-specific regulation for A-type RIFINs. Phylogenetic analysis of 5′-upstream regions showed that the *rif*–gene family falls into five defined clusters, designated rups (*rif* upstream) A1, A2, AB, B and C. In trophozoites and early gametocytes, *rif* variants of the rupsA2-type were preferentially expressed.

**Conclusions/Significance:**

In this work we demonstrate the expression dynamics of the *rif*-gene family during sexual differentiation and present indications for subgroup specific regulation patterns. Therefore, our data provide a first foundation and point to new directions for future investigations of the potential role of RIFINs in gametocyte immune evasion.

## Introduction

The protozoan parasite *Plasmodium falciparum* induces the most severe form of malaria and continues to be one of the leading causes of death worldwide, with children under the age of five years and pregnant women being most affected by the disease [1]. The life cycle of the malaria parasite is complex and comprises both an asexual and a sexual phase. In the human host, the rapidly asexually reproducing intraerythrocytic parasites are responsible for the malaria-associated morbidity and mortality. Sexual stage parasites, in contrast, facilitate the transmission from the human host to the mosquito vector. Considering their vital importance for the maintenance of the *Plasmodium* population, gametocytes are regarded as interesting targets for control measures aiming to reduce parasite transmission [2], [3].

Gametocytes may be male or female parasites, referred to as micro- and macrogametocytes, respectively. Gametocytogenesis takes about 10 days *in vitro* from the invasion of a sexually committed merozoite into a red blood cell until maturity is reached [4], [5]. During their differentiation, gametocytes undergo massive ultrastructural changes and five major phenotypically distinct stages (stages I–V) have been classified [6]. According to this classification, stage V gametocytes represent the population of fertilization-competent parasites that upon ingestion by a mosquito transform to male and female gametes, finally fusing into a zygote.

Despite their crucial position in the *Plasmodium* life cycle and their presence in the infected human for several days, it is not yet understood how gametocytes evade the host immune response. Similar to asexual parasites, immature stage I to IV gametocytes can leave the circulation and sequester at various sites in the human body such as the bone marrow and the spleen [7], [8]. Consequently, only mature gametocytes are present in the circulation. It is unclear how this is achieved, as knob structures associated with cytoadherence of asexual parasites appear to be present only in very young sexual parasites at stages I and II, but are absent later during maturation [9], [10].

Several host receptors implicated with adhesion of gametocytes have been found [10]–[13]. Still, parasite ligands mediating such interactions have not been unequivocally identified. Candidate proteins exposed at the surface of the gametocyte infected erythrocyte (IE) include modified band 3 as well as members of the variant antigen families present in the *P. falciparum* genome [13], [14]. In particular, PfEMP1 molecules, encoded by the *var* gene family and known to mediate cytoadhesion to many different receptors in asexual parasites [15], [16], have been suggested to function as gametocyte ligands for CD36 in early gametocytes [10]. However, it is currently considered unlikely that PfEMP1 molecules play a major role in sequestration of later immature gametocyte stages [14], [17], although specific members of the *var* gene family are selectively transcribed at low levels throughout gametocyte differentiation [18]. Apart from PfEMP1, members of a second family of variant molecules, the STEVORs, are known to be expressed in gametocytes and have been suggested to play a role in gametocyte immune evasion [18]–[20]. The mechanisms by which STEVOR proteins may contribute to this process however remain an enigma.

With respect to immune evasion of gametocytes, no attention has so far been paid to the third and largest of the protein families in *P. falciparum*, the RIFIN family. The genes encoding RIFIN proteins are mainly found in the subtelomeric regions of all 14 *P. falciparum* chromosomes. Here, they are clustered together with the *var* and *stevor* genes [21]. RIFINs and STEVORs share a similar protein architecture, both containing a semiconserved and a variable domain as well as a PEXEL/HT motif [22], [23], which labels them as exported proteins. Their large number and hypervariability support a role in antigenic variation. Recent evidence shows that RIFINs can be subdivided into two subgroups, A- and B-type RIFINs, which differ from each other by the presence or absence of 25 amino acids in the semi-conserved domain [21], [24], [25]. Furthermore, while A-type RIFINs appear to be exported via Maurer's clefts (MC) to the erythrocyte, B-type RIFINs seem to accumulate mainly inside the parasite. In contrast to PfEMP1, the functional relevance of STEVORs and RIFINs during the erythrocytic life cycle is not well established [26].

Until recently, RIFINs were only considered antigens of asexual blood stage parasites. A notable observation from transcriptome- and proteome-wide expression analyses is a particularly high rate of RNA and protein expression from variant antigen families in gametocytes and sporozoites [27], [28]. The heterogeneity in RIFIN expression detected in these high-throughput studies, as well as our observation that different RIFIN variants show divergent expression profiles and are developmentally regulated [24], inspired us to take a closer look at RIFIN expression during sexual differentiation of *P. falciparum*. Here, biochemical, genetic and phylogenetic tools are applied to investigate RIFIN expression during gametocytogenesis and to compare the expression patterns with those found in asexual parasites.

## Results

### RIFIN proteins show diverse developmental expression patterns during gametocytogenesis

**Figure 1:**
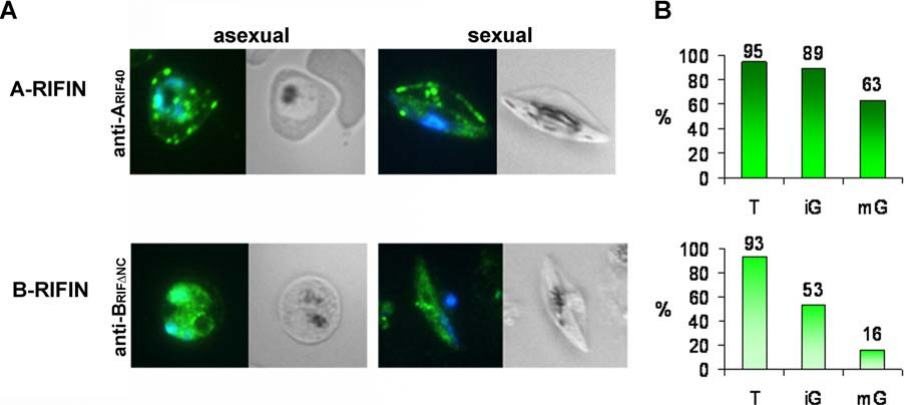
Immunofluorescence analysis of RIFIN expression in asexual and sexual parasites. A: Smears of trophozoite and gametocyte IE were analyzed for RIFIN localization by staining with rat anti-A_RIF40_ and mouse anti-B_RIFΔNC_ antisera (green). Nuclei were stained with DAPI (blue). A-type RIFINs were visualized with anti-A_RIF40_, while B-type RIFINs were detected with anti-B_RIFΔNC_. B: Quantification of the proportion of RIFIN positive cells in trophozoites (T), immature gametocytes at day 6 (iG) and mature gametocytes at day 10 (mG) after induction of gametocytogenesis.

To examine RIFIN expression in gametocytes, IFA was performed on methanol fixed smears of pigmented trophozoite infected erythrocytes (IE) as well as on gametocyte preparations harvested at day 6 after induction of gametocytogenesis, when the majority of the cells consisted of immature sexual stages III and IV (Table 1). The anti-A_RIF40_ and anti-B_RIFΔNC_ antisera directed against representative A and B-type RIFIN variants, respectively, were used as tools. In keeping with previous results [24], A-type RIFINs were exported to the host cell in asexual parasites and associated with the Maurer's clefts (MC), while B-type RIFINs were located mainly intra-parasitically (Fig. 1A, 1^st^ panel). In gametocytes, the subcellular localization of RIFINs resembled the one found in trophozoites, in that A-type RIFINs detected with anti-A_RIF40_ were distributed in dotted structures within the host cell, whereas B-type RIFINs labeled with anti-B_RIFΔNC_ accumulated inside the parasite (Fig. 1A, 2^nd^ panel). An estimation of the fluorescence rates revealed that both subtypes were detected in over 90% of the trophozoite IE population. In contrast, the proportion of positive cells was around 89% and 53% with anti-A_RIF40_ and anti-B_RIFΔNC_, respectively, in immature stage III–IV gametocytes (iG) harvested at day 6 after induction of sexual differentiation. During maturation to stage V (mG), the levels of labeled gametocytes decreased to 63% with anti-A_RIF40_ and 16% with anti-B_RIFΔNC_ at day 10 after induction (Fig. 1B).

**Table 1:** Proportion of asexual and sexual parasite stages during gametocyte culture.

	% of parasite stages*					
	Trophozoites	Gametocytes				
		Stage I	Stage II	Stage III	Stage IV	Stage V
Day 0	**94,5±3,1**	2,7±1,2	2,7±2,3	0	0	0
Day 2	0	**86,5±3,2**	13,5±3,2	0	0	0
Day 4	0	1,9±0,02	**80,5±3,1**	17,5±3,2	0	0
Day 6	0	0	3,3±2,1	**88,8±3,5**	8±1,7	0
Day 8	0	0	0	6,1±1,2	**76,4±3,2**	17,6±2,7
Day 10	0	0	0	3,5±1,6	18,8±4,3	**77,7±5,4**

* Parasite counts were performed on thin smears of parasite culture. The mean and standard deviation were calculated from three experiments.

**Figure 2:**
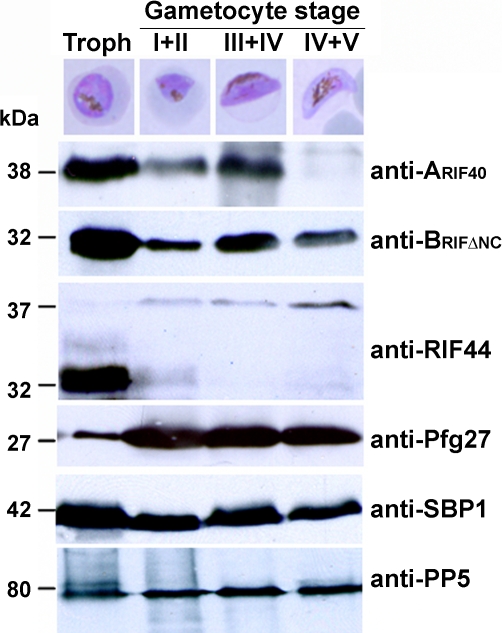
Western Blot Analysis of RIFIN expression during gametocytogenesis. Total lysates from trophozoite and gametocyte IE at different time points during sexual development (stages shown representatively in the top panels) were analyzed using antisera generated against recombinant RIFIN proteins RIF40, RIFΔNC and RIF44. The antiserum against the gametocyte specific protein Pfg27 served as a stage control. Antibodies against the Maurer's clefts marker protein SBP1 and the soluble parasite protein phosphatase PP5 served as loading controls. Molecular size markers in kDa are indicated on the left.

To substantiate the presence of RIFINs in gametocytes, lysates of MACS enriched trophozoites, stages I and II (day 2), stages III and IV (day 6) or mainly mature stage V (day 10) gametocytes were analyzed by western blotting (Fig. 2). Protein bands of 38 and 32 kDa were detected in all fractions with anti-A_RIF40_ and anti-B_RIFΔNC_, respectively. The intensity of both protein bands in early stages I and II as well as in more mature stages IV and V was markedly reduced in comparison to asexual or intermediate sexual parasite stages. This observation was interpreted as a demonstration that peak expression of RIFIN variants detected with anti-A_RIF40_ and anti-B_RIFΔNC_ occurred in stage III and IV gametocytes, as supported by the fluorescence rates observed by IFA (Fig. 1B). Interestingly, when testing a third anti-RIFIN antiserum directed against a recombinant A-type RIFIN (RIF44) [29], two protein bands of 32 and 37 kDa were apparent (Fig. 2). While the 32 kDa protein was exclusively expressed in asexual parasites, the 37 kDa protein was first observed in early gametocytes with an increasing intensity during maturation. Although this result clearly demonstrates that the RIFIN pool in gametocytes differs from the one in asexual parasites, it is unknown whether the two protein bands derive from differentially processed or otherwise modified types of the same RIFIN, or whether they represent distinct variants. The gametocyte specific antigen Pfg27 [30] was used as a stage control and was found to be highly expressed in all gametocyte fractions. Additionally, the transmembrane protein SBP1 [31] as well as the soluble protein protein phosphatase 5 (PP5) [32] were used as loading controls.

### Subcellular localization of RIFINs indicates host cell membrane-association of A-type variants in gametocytes

Elucidation of the subcellular compartments harboring RIFINs in gametocytes might help to uncover the functional relevance of these variant antigens during sexual development. Therefore, colocalization studies were performed to visualize the RIFIN distribution in relation to subcellular marker proteins of known locations. Since it was shown above that gametocytes exhibited maximal protein expression of RIFIN variants detected with anti-A_RIF40_ and anti-B_RIFΔNC_ in the stages III and IV collected around day 6 of gametocytogenesis, methanol fixed smears of cultures at this time point were chosen for analysis.

**Figure 3:**
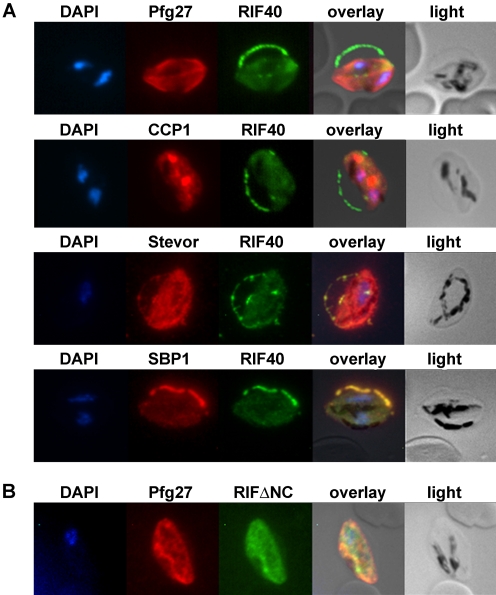
Immunofluorescence colocalization in stage III gametocytes. Methanol fixed smears of stage III gametocytes were incubated with antibodies against RIFIN and marker proteins. A: rat anti-A_RIF40_ antibodies (green) and rabbit anti-Pfg27, mouse anti-CCP1, mouse anti-STEVOR or mouse anti-SBP1 (red). B: mouse anti-B_RIFΔNC_ (green) and rabbit anti-Pfg27 (red). Nuclei were stained with DAPI (blue).

Double staining experiments using an antiserum directed against the cytoplasmic parasite protein Pfg27 [30], [33] together with the anti-RIFIN antisera were performed. Signals observed within the parasite using anti-A_RIF40_ and antiserum against Pfg27 only faintly overlapped with each other (Fig. 3A, 1^st^ row). Instead, the A-type RIFIN related fluorescence was largely concentrated in a “string of pearls” pattern at the erythrocyte membrane. Here, A-type RIFINs detected with anti-A_RIF40_ apparently accumulate in the tubular space defining the fringe of the hemoglobin-depleted erythrocyte apron, referred to as Laveran's bib. In contrast, B-type RIFIN specific staining with anti-B_RIFΔNC_ overlapped largely with Pfg27 (Fig. 3B).

We investigated whether the punctate fluorescence detected with anti-A_RIF40_ antiserum colocalized with the recently discovered LCCL domain-containing family protein PfCCP1. PfCCP1 has been described to display a discretely dotted appearance in IFA and shown to be exported to the parasitophorous vacuole (PV) in gametocytes [34], [35]. Since the fluorescence patterns of A-type RIFINs and PfCCP1 did not appear to coincide with each other, a PV restricted localization of A-type RIFINs is considered to be unlikely (Fig. 3A, 2^nd^ row).

The staining of A-type RIFINs in gametocytes was also examined in relation to variant antigens of the STEVOR family, which have been reported to be present at the host cell membrane in gametocytes [19]. Anti-STEVOR fluorescence indeed overlapped with that of anti-A_RIF40_ at the erythrocyte membrane and in MC like compartments (Fig. 3A, 3^rd^ row). Moreover, colocalization of the anti-A_RIF40_ signal with that of the well-characterized MC protein SBP1 suggested A-type RIFIN localization in these structures (Fig. 3A, 4^th^ row). These staining patterns indicated that all three proteins are located in the same subcellular compartment, although this contradicts the previous suggestion that STEVORs reach the erythrocyte surface in gametocyte IE via a MC-independent pathway [19].

### *Rif* transcripts are detected throughout sexual differentiation

The analysis of RIFIN expression on the protein level using crossreactive antisera has its limitations in assigning the exact identity to the detected variants. To overcome this problem and to address the developmental changes in *rif* gene expression, RNA transcription profiles were determined by RT-PCR.

**Figure 4:**
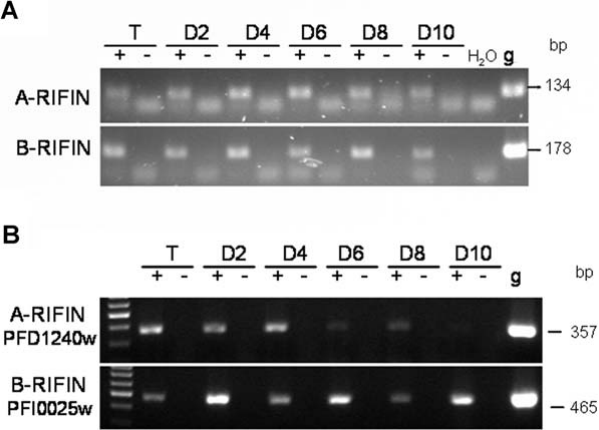
RIFIN RNA expression during sexual development. A: RT-PCR analysis of A- and B-type RIFINs in asexual and sexual stage parasites of the NF54 strain. RNA was harvested from 20–24 hour trophozoites (T) and at days 2, 4, 6, 8 and 10 of gametocytogenesis (D2–D10). RNA was reverse transcribed into cDNA (+) and PCR was performed with specific primers for A-RIFINs and B-RIFINs. RNA samples in which reverse transcriptase was omitted served as negative controls (−). Positive controls were performed on genomic DNA (g). B: RT-PCR analysis with specific primers detecting the A-type *rif* variant PFD1240w and the B-type *rif* variant PFI0025c. Stages are as in (A).

RNA was isolated from trophozoites 20–24 hours post invasion as well as from day 2, 4, 6, 8 and 10 gametocyte cultures and subsequently reverse transcribed into cDNA and amplified with the help of the degenerate primer pairs RIFAFor/Rev and RIFBFor/Rev [24]. While gDNA served as a positive control, samples in which reverse transcriptase was omitted during cDNA synthesis served as negative controls. Products of the predicted sizes of 134 bp and 178 bp for A- and B-type RIFINs, respectively, were amplified from gDNA as well as from all cDNA samples of asexual and sexual stage parasites, but not from the negative controls (Fig. 4A). This result was taken as an indication that both A- and B-type RIFIN expression proceeded throughout sexual development. The presence of full length *rif* transcripts was verified by northern blot analysis of RNA samples from young trophozoites and days 2, 6 and 10 gametocytes, indicating that PCR products were not the result of promiscuous or aborted transcription (data not shown).

### Developmental expression patterns of A- and B-type *rif* variants

**Figure 5:**
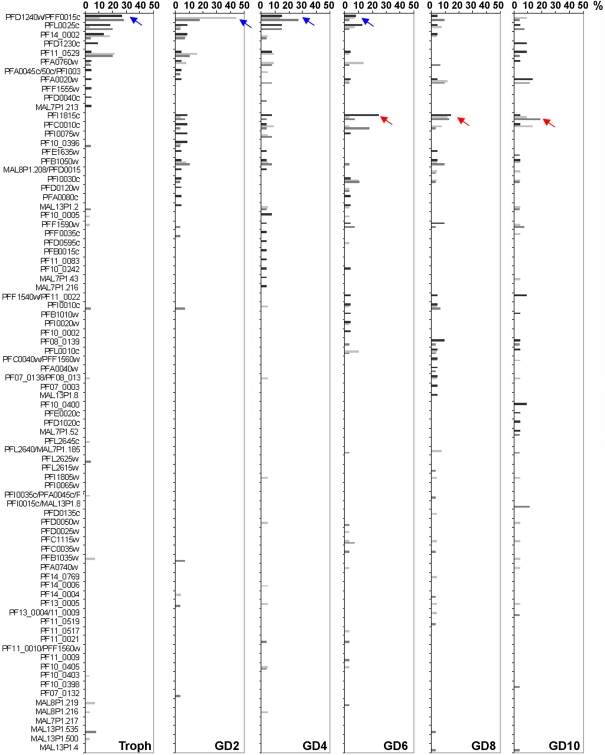
Changes in A-type *rif* gene transcription during sexual differentiation. A-type *rif* RT-PCR products from 20–24 hour trophozoites (Troph) as well as day 2, 4, 6, 8 and 10 gametocytes (GD2–GD10) were cloned and single sequences analyzed. Frequencies are shown in percent of all clones. Blue arrows highlight downregulation, red arrows upregulation during sexual development. Three independent experiments are shown in black, light grey and dark grey.

**Figure 6:**
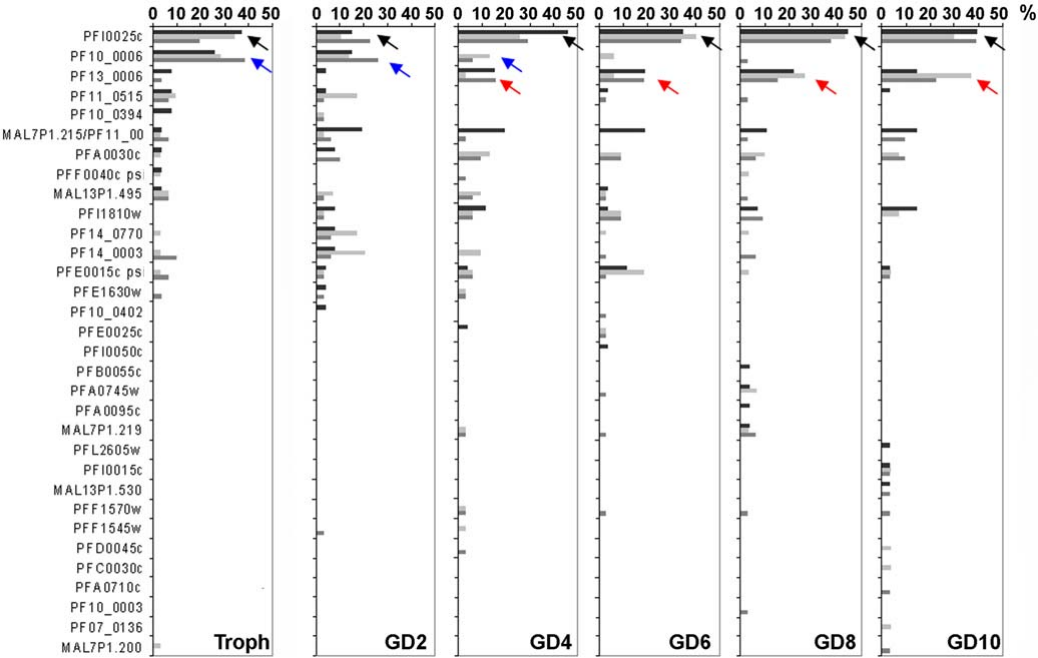
Changes in B-type *rif* gene transcription during sexual differentiation. B-type *rif* RT-PCR products from 20–24 hour trophozoites (Troph) as well as day 2, 4, 6, 8 and 10 gametocytes (GD2–GD10) were cloned and single sequences analyzed. Frequencies are shown in percent of all clones. Black arrows point out variants that are continuously detected, blue arrows highlight downregulation, red arrows upregulation during sexual development. Three independent experiments are shown in black, light grey and dark grey.

Extending this analysis, nucleotide sequences of the RT-PCR products obtained from three independent experiments were further examined. A- and B-type RIFIN RT-PCR products from each developmental stage were cloned and single colonies were analyzed by sequencing. Results from one representative experiment are shown in Figs. 5 and 6 for A- and B-type RIFINs, respectively. Unbiased amplification of different *rif*-variants was verified by analysis of gDNA clones. In 20 clones, 15 and 14 different sequences were detected with A- and B-type RIFIN specific primer pairs, respectively. All sequences occurred with similar frequencies, none of them more than twice (data not shown). Some variants were found to be identical over the analyzed stretch, thereby preventing unequivocal assignment of these clones, as indicated in Figs. 5 and 6.

Analysis of A-type cDNA clones revealed that dominant variants (defined to occur with a frequency of >25%) were only detected in two out of three experiments in trophozoites, and in one out of three experiments in day 2, 4 or 6 gametocytes, respectively (Table 2, Fig. 5). Generally, a broad range of different A-type *rif* variants was detected in each experiment at similar frequencies, indicating that there is no clear dominance of single variants in any of the stages (Fig. 5, Table 2). Noteworthy, however, was the finding that certain sequences were repeatedly detected more frequently in trophozoites and early gametocytes than in late gametocyte stages (e.g. PFD1240w/PFF0015c, blue arrow in Fig. 5), whereas others were found exclusively in gametocyte stages in all three experiments (e.g. PFI1815c, red arrow). This observation may indicate the existence of stage specific regulation mechanisms for different A-type variants.

**Table 2:** Summary of A-type *rif* clones.

	Experiment 1°		Experiment 2°		Experiment 3°	
	No. of Sequences/Clones#	Dominant sequence* (frequency)	No. of Sequences/Clones#	Dominant sequence* (frequency)	No. of Sequences/Clones#	Dominant sequence* (frequency)
Troph	11/23	PFD1240w/PFF0015c (27%)	16/28	–	10/25	PFD1240w/PFF0015c (28%)
GD2	17/23	–	10/25	PFD1240w/PFF0015c (44%)	16/28	–
GD4	17/26	–	17/22	–	13/26	PFD1240w/PFF0015c (27%)
GD6	16/24	PFI1815c (25%)	20/29	–	19/28	–
GD8	17/21	–	19/26	–	20/31	–
GD10	16/22	–	18/22	–	16/26	–

° A-type RIFINs.

* frequency >25%.

# Number of different sequences detected/ number of clones analyzed.

In contrast to A-type RIFINs, the expression patterns observed for B-type RIFIN variants were highly consistent between all three experiments and indicated strict regulation during sexual differentiation (Table 3, Fig. 6). PFI0025c transcript (black arrow in Fig. 6) was detected in all preparations at all stages and was the dominant transcript in trophozoites (2/3 experiments) and in day 4 to day 10 gametocytes (3/3 experiments). PF10_0006 (blue arrow) was always detected at high frequency in trophozoites, but not in gametocytes, representing a B-type *rif* variant preferentially expressed in asexual stages. PF13_0006 (red arrow), in turn, clearly became more abundant in late gametocytes, although it was dominant only in one experiment. Interestingly, in two of the three experiments no dominant transcript was detected in day 2 gametocytes. In individual experiments, single sequences showed similar tendencies in regulation as the mentioned variants, although none was found at dominant levels.

**Table 3:** Summary of B-type *rif* clones.

	Experiment 1°		Experiment 2°		Experiment 3°	
	No. of Sequences/Clones#	Dominant sequence* (frequency)	No. of Sequences/Clones#	Dominant sequence* (frequency)	No. of Sequences/Clones#	Dominant sequence* (frequency)
Troph	9/27	PFI0025c (37%)	11/32	PFI0025c (34%)	9/31	PF10_0006 (39%)
		PF10_0006 (26%)		PF10_0006 (28%)		
GD2	12/26	–	10/29	–	13/31	PF10_0006 (26%)
GD4	6/26	PFI0025c (46%)	12/31	PFI0025c (26%)	13/31	PFI0025c (29%)
GD6	8/26	PFI0025c (35%)	9/32	PFI0025c (41%)	13/32	PFI0025c (34%)
GD8	8/27	PFI0025c (44%)	8/30	PFI0025c (43%)	12/32	PFI0025c (38%)
				PF13_0006 (27%)		
GD10	9/28	PFI0025c (39%)	9/27	PFI0025c (30%)	10/31	PFI0025c (39%)
				PF13_0006 (37%)		

° B-type RIFINs.

* frequency >25%.

# Number of different sequences detected/number of clones analyzed.

RT-PCR using specific primers confirmed predominant expression of the A-type variant PFD1240w in trophozoites and early gametocytes as well as expression of the B-type variant PFI0025c in all analyzed stages (Fig. 4B).

### Analysis of non-coding upstream regions allows definition of further RIFIN subgroups

The above analysis of single A- and B-type RIFIN variants did not enable us to draw a clear picture especially of the regulation of A-type RIFIN expression during *P. falciparum* development. It is known that the developmental expression of genes is often regulated by elements which are present in the 5′-upstream regions flanking a protein coding gene [36], [37] and several studies have demonstrated that sequence information in the 5′-upstream regions of *P. falciparum* genes is necessary and sufficient to drive stage specific transcription [38]–[44]. Therefore we sought to characterize the corresponding sequences of *rif* genes with the aim to identify motifs potentially related to stage specific transcription. This was further encouraged by a recent publication indicating that *var* genes under the control of a 5′-upstream region belonging to the upsC type are preferentially expressed in gametocytes [18].

Joannin et al. recently performed a phylogenetic analysis of 500 bp of the 5′-upstream region of the *rif* genes present in the 3D7 genome [25]. However, it is known that *P. falciparum* genes have unusually long 5′-UTRs (untranslated regions) [45]. Moreover, although the transcriptional start site has been mapped to position −245 from the start codon for one *rif* gene, it was shown that negative regulatory elements are present further upstream of the transcription start site up to position −870 [46]. Similarly, putative regulatory elements present in the upstream region of *var* genes have been identified as far as 1426 bp preceding the translation start site [47]. Based on this information, 1.5 kb of the noncoding 5′-upstream regions were incorporated into our analysis. A distance tree was inferred from an alignment of 140 *rif* upstream regions using the p-distance/Neighbor-Joining method. Gaps were treated as pairwise deletions. To test the reliability of the classification, 500 bootstrap replications were performed.

**Figure 7:**
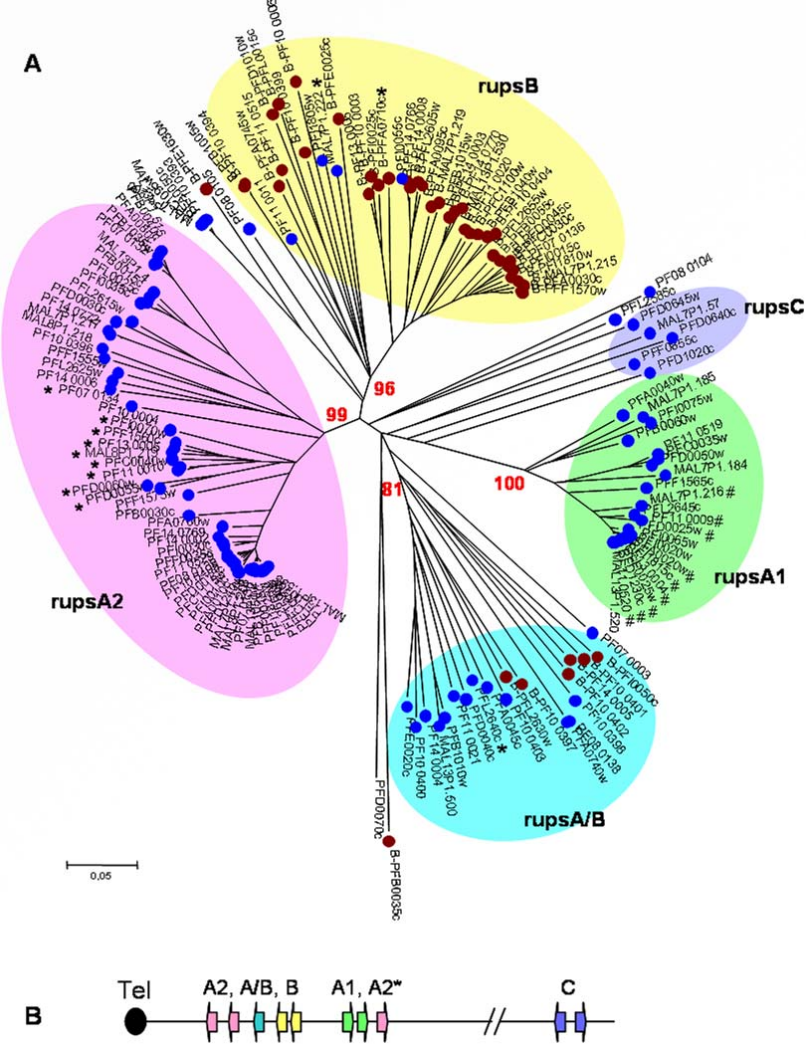
Definition of *rups* groups based on 5′-upstream sequences. A: Distance tree of *rif* gene upstream regions (rups) from 3D7. The 5′-sequences containing 1500 bp upstream of each *rif* gene were aligned with ClustalW. Distance trees were generated using the p-distance/Neighbor-Joining method with pairwise deletion of gaps. The clusters rupsA1, rupsA2, rupsA/B and rupsB were verified by bootstrapping, and bootstrap values are indicated at the branches. The cluster rupsC could not be verified by bootstrapping. Star: RupsA2, rups A/B and rupsB variants with transcription orientation towards centromere, contrary to general transcription orientation. Hash: Upstream regions previously referred to as upsA-*rif* [48]. B: Chromosomal orientation of variants belonging to each of the rups clusters.

The resulting tree revealed the existence of five major *rif* upstream groups, in the following referred to as “rups” (for *rif* upstream) (Fig. 7A). Two large sequence clusters contained exclusively A-type *rif* gene upstream sequences and were named rupsA1 (20 variants) and rupsA2 (49 variants), the latter further segregating into two smaller groups. Interestingly, all rupsA1 related sequences show a transcription orientation towards the centromere, whereas the majority of A2 related variants are transcribed towards the telomere with the exception of nine variants (Fig. 7A stars, Fig. 7B, Suppl. Table S1). Noteworthy, the rupsA1 cluster comprises the sequences that were previously referred to as upsA-*rif* because they are flanked by the 5′-upstream regions of an adjacent *var* gene of the upsA type transcribed in the opposite direction [48] (Fig. 7A hash, Suppl. Table S1).

One sequence cluster consisted mainly of B-type *rif* gene upstream sequences and is thus referred to as rupsB (38 variants). In this cluster, four upstream sequences belonging to A-type *rif* genes were present. Another cluster contained both A- and B-type sequences and was called rupsA/B (19 variants). Both, rupsA/B and rupsB variants generally display an orientation directed towards the telomere (Fig. 7B, Suppl. Table S1) with the exception of one and two genes, respectively (Fig. 7A star). A very small fifth cluster harboring A-type variants was evident, which according to the long branches contained rather divergent 5′-upstream sequences, and was named rupsC (5 variants). Examination of the chromosomal location of rupsC related variants revealed that these are the only variants located in proximity to the centromeres. Bootstrapping showed that all definitions except for rupsC were highly reliable. Various single sequences (9 variants) did not group with any of the rups clusters.

In summary, a good correlation between the 5′-upstream sequences and the genomic orientation was observed in agreement with an earlier analysis of *rif* gene upstream regions [25], encouraging the new classification of *rif* genes. However, examination of the records from transcriptomic and proteomic studies for each *rif* gene did not allow us to draw any conclusions concerning stage-specific expression related to any of the rups groups (Suppl. Table S1).

To verify the presence of the rups clusters in other *P. falciparum* strains, we examined 1000 bp of the upstream sequences of *rif* genes annotated in the HB3 genome in an alignment with the corresponding 3D7 sequences. We took advantage of a previously published phylogenetic analysis of the HB3 *rif*-coding sequences for the selection of A- and B-type *rif* genes [25]. Of these genes, 53 5′-upstream sequences were available, only seven of which belonged to B-type RIFINs. Despite the scarcity of valid B-type *rif* sequences, all rups clusters could be verified in the resulting tree (Suppl. Fig. S1).

### Subgroup specific *rif* gene expression during gametocytogenesis in view of rups classification

**Figure 8:**
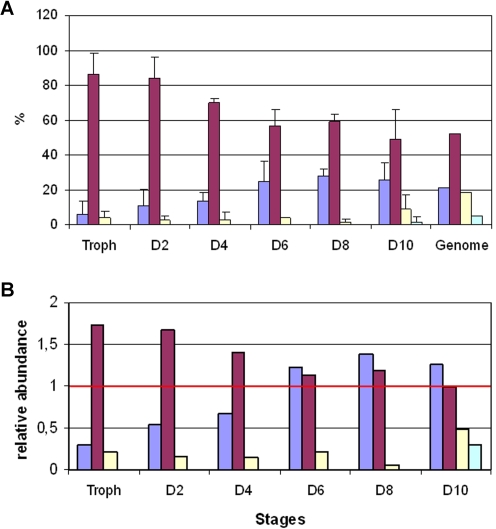
Rups related A-type *rif* variant expression in asexual and sexual stages. A: Proportion of A-type variants summarized by rups group in asexual and sexual stages. The mean and standard deviation of three independent experiments is shown. B: The frequencies of variants assigned to the rups groups in each stage were corrected for the frequency of variants of the respective rups group in the genome. A relative abundance value of 1 corresponds to the identical proportion as present in the 3D7 genome. A1: blue, A2: red, AB: yellow, C: green.

To examine the contribution of variants with specific 5′-upstream sequences to the *rif* transcriptomes of asexual and sexual parasites, we sorted the cloned A-type *rif* sequences from above according to their allocated rups groups and compared the abundance of each type in the different parasite stages. In all stages, the majority of clones belonged to the rupsA2 type, and this was most obvious in asexual and early sexual developmental stages (Fig. 8A). However, because genes related to the different rups groups are present in unequal numbers in the *P. falciparum* genome, we corrected for their respective gene numbers (Fig. 8B). Strikingly, in trophozoites and early gametocytes (day 2 to 4), clones representing variants of the rupsA2 type were significantly overrepresented, whereas clones belonging to the rupsA1 or rupsA/B RIFIN groups were underrepresented. In day 6 to 10 gametocytes, however, the proportion of A-type variants belonging to each rups-type resembled approximately their frequency in the genome (relative abundance value of 1) indicating no significant preference in expression of any rups-type.

In all stages, the dominant B-type variants belonged to the rupsB group and rupsA/B related transcripts were underrepresented (data not shown).

## Discussion

We present here the first analysis of RIFIN expression dynamics during gametocytogenesis. Our data suggest the following conclusions. Firstly, RIFIN expression occurs in multiple stages including asexual and sexual parasites, and RIFIN variants show diverse developmental regulation patterns. Secondly, supported by their distinct subcellular localizations, A- and B-type RIFINs probably exert different biological functions. Thirdly, regulation of A- and B-type *rif* genes appears to be uncoupled from each other, as evidenced by the finding of high conservation in developmental expression of single B-type RIFIN variants, while this is not the case for A-type RIFINs. In support of this, A- and B-type *rif* genes possess distinct non-coding upstream regions. Finally, expression of variants falling into the rupsA2 cluster seems to dominate in trophozoites and early gametocytes, an observation which may indicate that A-type RIFIN variants are transcribed in a group-specific fashion, whereas B-type RIFINs appear to be regulated on the level of single variants.

To date, the biological function of RIFINs is not clear. An involvement in antigenic variation and immune evasion is strongly supported by the presence of a hypervariable region and the existence of a large repertoire of more than 150 *rif* genes in the genome of a single parasite. Proteome- and transcriptome-wide analyses have indicated that RIFIN expression is not restricted to asexual intraerythrocytic parasites, but also occurs in the invasive parasite stages such as merozoites and sporozoites, as well as in gametocytes [27], [28]. The same holds true for members of other variant antigen families. Experimental evidence supporting expression of STEVOR, PfEMP1, SURFIN and RIFIN proteins across various stages is accumulating [14], [18], [19], [24], [49], [50], and is extended in the present work by the demonstration of RIFIN expression in gametocytes. The phenomenon of multistage expression of multigene families is not restricted to *P. falciparum*, but has also been described in the rodent malaria parasite *P. yoelii*, where different repertoires of the py235 gene family are expressed in erythrocytic or hepatic merozoites and sporozoites [51]. Similarly, variants of the *P. berghei* BIR family have been identified in erythrocytic, mosquito and sporozoite stages in proteomic studies [52]. In the apicomplexan parasite *Toxoplasma gondii* developmental expression of subsets of variant surface antigens of the SRS superfamily is known in tachyzoites and bradyzoites [53], [54].

Given that all stages present in the human host are potentially exposed to the immune system and consequently need to develop mechanisms to circumvent recognition and clearance, multi-stage expression of variant antigens in *Plasmodium* species would be beneficial to parasite survival. Gametocytes persist in the human host for several days and have been reported to sequester in the bone marrow during maturation [7]. Yet few data are available on the underlying mechanisms as to how they evade host immunity over an extended period. Members of the PfEMP1 family of variant antigens have been located only at the surface of very early stage I and II gametocytes, where they are thought to act as ligands for the host receptor CD36 [14]. However, CD36-mediated cytoadhesion ceases during sexual differentiation and switches to a cytoadhesive phenotype, which can be reversed by antibodies directed against the human antigens CD49c, CD166 and CD164 present on human bone marrow epithelial and stromal cells [11]. Variants belonging to the RIFIN-related protein family of STEVORs have been reported to be present on the host cell surface until maturity of gametocytes [19]. However, a role in cytoadhesion remains speculative and is deemed unlikely, simply because mature stage V gametocytes do not sequester. Thus, a parasite ligand mediating cytoadhesion of intermediate gametocytes of stages III and IV in the bone marrow still remains unknown. In fact, RIFINs, previously implicated in rosette formation between IE and uninfected red blood cells [55], [56] and adhesion to CD31 [57], represent good candidates as mediators of cytoadhesive interactions of gametocytes.

Our demonstration here of A-type RIFINs at the IE membrane in intermediate sexual stage parasites provides a solid basis for investigating their involvement in cell-cell interactions in the bone marrow. The observed “string of pearls” pattern along the membrane of the hemoglobin depleted erythrocyte agrees with possible surface exposure. Moreover, localization of A-type RIFINs overlapped with that of STEVOR and the MC marker protein SBP1, thereby reinforcing that A-type RIFINs are trafficked across the PV membrane towards the membrane of the infected cell [19], [31]. Nevertheless, direct evidence of surface exposure in the gametocyte IE is still lacking.

A recent study analyzed recognition of trophozoite and gametocyte IE by sera from *P. falciparum* infected children [58]. Interestingly, there was no correlation between the capacity to react with the surface of asexual parasite IE and mature sexual parasite IE, indicating that surface antigens might differ significantly between the two developmental stages. Moreover and rather unexpectedly, the study showed that although mature stage V gametocytes and asexual parasites were frequently recognized, antibodies against immature gametocyte stages were rarely detected. This argues for a very low antigenic profile of immature gametocytes and supports a mechanism by which sequestration in the bone marrow renders the parasites inaccessible to immune recognition. RIFINs have previously been shown to be immunogenic in ELISA studies [29], [59], however, their hyper-variability might explain the lack of surface recognition in the FACS analyses of immature stages presented by Saeed et al [58].

An intriguing finding in our present and previous study [24] is the subcellular localization of B-type RIFINs, which is found in the parasite compartment labeled with Pfg27 in gametocytes, disagreeing with the common conception of RIFIN involvement in antigenic variation at the surface of infected cells. Recently, Lavazec et al. proposed a model according to which RIFINs, STEVORs and members of the PfMC2TM family of proteins might contribute to the formation of ion channels in the membrane [60]. The conserved expression patterns in three different cultures as shown by transcriptional analysis and the reduced variability [25] support the finding that B-type variants are not exported to the host cell surface. Instead, they might exert an analogous function as a channel molecule at the parasite or PV membrane.

When analyzing A- and B-type RIFIN expression in different stages during the *P. falciparum* life cycle, we found that *rif* transcription, unlike *stevor* transcription [19], seems to occur continuously during gametocyte differentiation. The detection of RIFIN transcripts throughout gametocytogenesis is in contrast to the tight temporal control of *rif* genes in early trophozoites during asexual differentiation [61]. However, on the protein level, distinct patterns of developmental regulation were apparent for single variants. Both the A-type specific antiserum anti-A_RIF40_ and the B-type specific antiserum anti-B_RIFΔNC_ detected variants in both trophozoites as well as in gametocytes with peak expression at stage III/IV. In contrast, a third anti-RIFIN antiserum, anti-RIF44, reacted with two distinctly regulated variants, one of which was upregulated during gametocytogenesis while expression of the other ceased.

Although subtype-restricted recognition has previously been validated for anti-A_RIF40_ and anti-B_RIFΔNC_ [24], this has not been shown for anti-RIF44. Moreover, the exact specificity of the antibodies for variants of the NF54 RIFIN repertoire is not known. It is likely that only a limited number of similar RIFIN variants are detected, making it difficult to draw general conclusions on RIFIN expression patterns during gametocytogenesis, as relevant proteins may not be visualized and assignment of the variant is difficult. However, in support of our protein related results similar differential stage specific expression patterns of RIFIN variants were found and further specified on the RNA level in RT-PCR experiments.

Noteworthy is the high reproducibility of transcription patterns found for B-type RIFINs in three independent experiments, with exactly the same variants dominantly expressed at different time points during differentiation. In contrast, the A-type RIFIN repertoire changed significantly between the three experiments and dominant variants were rarely detected. Comparing our data with that obtained by a microarray approach to analyze expression profiles of asexual parasites and developing gametocytes of the 3D7 and NF54 strains [5], it was interesting to observe the same patterns of developmental expression for RIFINs in NF54. More specifically, the B-variant PF10_0006 was expressed in early trophozoites, and expression during gametocytogenesis was only detectable until day 2. Similarly, while expressed at very low level in asexual parasites, transcription of the B-variant PF13_0006 was upregulated five-fold in intermediate gametocytes. Similar tendencies in both studies are also obvious for A-type variants, although expression of these *rif* genes occurred at lower level in gametocytes. The concordance between both studies thus validates our experimental approach.

Recently, a study proposed a sexual stage specific program of *var* transcription based on the analysis of *var* expression dynamics during gametocytogenesis in three phenotypically different asexual populations [18]. Although the lack of a method to select a parasite population homogeneous in expression of a certain *rif* gene currently makes it difficult to follow a similar approach, our data support variant-specific transcription of *rif*-genes in sexual stages.

Here, five new *rif*-gene subgroups were defined according to their 5′ upstream regions. The corresponding trees largely overlap with the phylogenetic analysis performed previously [25]. However, with the aim to pick up regulatory motifs present upstream of the transcription start sites our analysis extended the previous one by including 1500 bp sequences instead of 500 bp only. This allowed a more refined analysis and appeared to increase sensitivity as demonstrated by the high bootstrap values clearly differentiating four clusters instead of only two [25]. A group of *rif* genes earlier referred to as upsA-*rif* due to their head to head organization with adjacent *var* genes was found to cluster closely together in the rupsA1 group, supporting our classification [46], [48].

RT-PCR results support a more pronounced expression of rupsA2-type variants than rupsA1 or any other type in asexual and immature sexual stages, indicating differential regulation of the two gene clusters. A recent study addressed regulatory elements in the 5′-upstream region of a single *rif* gene [46] featuring an upsA-*rif* upstream region [48] and according to our novel classification belonging to the rupsA1 group. Two silencing elements could be identified. Interestingly, these silencing motifs appear to be conserved only in the rupsA1 cluster and in none of the other clusters, although they are present in single upstream sequences of other type (data not shown) as was already noted by others [25]. This strongly supports our finding of rups-group specific gene regulation and encourages the characterization of regulatory elements responsible for the expression or repression of the other *rif*-gene repertoires. In all rups clusters, conserved rups group-specific motifs are evident (not shown) which in the future can be analyzed with respect to putative regulatory functions.

Although our work clearly demonstrates the presence of RIFINs in *P. falciparum* gametocytes, their functional significance remains speculative. Our analysis has provided a selection of target genes for closer inspection of a potential role in cytoadhesion. Another open question concerns the interplay between proteins of the multigene families of *rif*, *stevor* and *var* genes and possibly other gene families undergoing multistage expression. In the light that certain variants belonging to the rupsA/B and rupsC clusters are markedly underrepresented in asexual and sexual IE, their expression in other parasite stages, such as sporozoites, could serve to be a promising line of investigation. In analogy to previous work demonstrating differential expression of *var* gene repertoires in isolates from patients with different clinical symptoms, the role of the different RIFIN groups in malaria pathology now awaits to be determined. In this respect, a detailed functional inspection of the impact of the distinct 3′-flanking sequences [25] on gene regulation might help to further narrow down repertoires of similarly regulated *rif*-genes.

## Materials and Methods

### Parasite strains and parasite culture

*Plasmodium falciparum* parasites of the NF54 line were used in this study and cultivated according to standard procedures in O+ erythrocytes [62]. Standard culture medium consisted of RPMI 1640 with L-glutamine (PAA) supplemented with 25 mM HEPES, 50 µg/ml Gentamycin, 0.5% Albumax II, 2% human AB+ serum and 0.005% Hypoxanthine. For gametocyte cultivation, a modified version of the protocol described by Fivelman et al. was applied [4]. Prior to induction of gametocytogenesis, parasites were synchronized by treatment with 5% sorbitol at ring stage and cultivated at a parasitemia of about 1% under standard conditions. Two days later, synchronized sexual development was stimulated by starving the fast-growing ring-stage culture at 6–8% parasitemia in the presence of partially spent medium. To do so, only one third of the medium was removed and replaced by fresh medium. Parasites were kept in culture for 32 h without further medium exchange. The culture was then split into five flasks and fresh medium and blood was added to obtain a hematocrit of 5%. The following day, when the cultures contained mainly ring-stage parasites, was considered day 0 of gametocytogenesis. The spent medium was removed and replaced by fresh medium containing 50 mM N-Acetyl-D-Glucosamine. Afterwards, the medium was exchanged every second day. Before harvesting parasites at various time points during gametocytogenesis, the parasite cultures were pooled, an aliquot taken and the remaining culture redistributed to culture flasks. Enrichment of gametocytes was achieved by MACS purification [63] and gametocyte stages assigned according to Carter and Miller [6].

### Antisera

The following antisera were applied in this study: rat anti-A_RIF40_ [29], mouse anti-B_RIFΔNC_ [64], rat anti-RIF44 [29], mouse anti-STEVOR [65] and rabbit anti-PP5 [32]. Mouse anti-SBP1 was kindly provided by Catherine Braun-Breton (Institute Pasteur, Paris), mouse anti-PfCCP1 was kindly provided by Gabriele Pradel (University of Würzburg), rabbit anti-Pfg27 was a kind gift from Pietro Alano (Istituto Superiore di Sanità, Rome).

Horseradish peroxidase (HRP) and Cy2 or Cy3 coupled secondary antibodies were purchased from Dianova. AlexaFluor coupled secondary antibodies were derived from Molecular Probes.

### Indirect immunofluorescence analysis (IFA)

Smears were prepared from parasite cultures at approximately 20% hematocrit. The smears were air dried and fixed for 5 min in 100% methanol at −20°C. Up to 8 fields were marked with a silicon pen (DakoCytomation). After rehydration for 10 min in PBS, the slides were incubated with antisera diluted in PBS/1% BSA (2 h at RT), washed 3 times with PBS, and incubated with fluorophore conjugated secondary antibodies and DAPI (1 µg/ml) for 2 h at RT. After repeated washing in PBS, the slides were embedded with MOWIOL (Serva), covered with a coverslip and analyzed with a 100× oil immersion lens in a UV equipped Leica DM RB microscope.

### Western blot analysis

Trophozoite and gametocyte IE were enriched by MACS. Total extracts were prepared by lysis in 2× SDS sample buffer at a concentration of 1×10^6^ IE/µl. Proteins corresponding to 1×10^7^ IE equivalents per lane were separated by SDS-PAGE and transferred to nitrocellulose membranes by semi-dry blotting. The transfer was performed for 1 h 20 min at 1.5 mA/cm^2^. Efficient protein transfer was controlled for by staining with Ponceau S solution and the membranes sequentially incubated with primary and secondary antibodies. Antigen detection was achieved with a chemiluminescence system (ECL, Amersham). To reanalyze the same samples with different antisera, the membranes were stripped and re-incubated.

### RNA isolation

Total RNA was extracted from *P. falciparum* IE essentially according to the protocol described by Kyes et al. [61] and resuspended in DEPC treated H_2_O for cDNA synthesis or in formamide for northern analysis. In order to minimize the risk of DNA contamination in RNA samples applied for cDNA synthesis and RT-PCR, an additional purification step using the *RNeasy Mini Kit* (QIAGEN) was conducted. The protocol was performed according to the instructions given by the manufacturer including the optional on-column DNA digestion step. RNA was eluted in 30 µl RNase free H_2_O and the eluate reloaded once to increase the final RNA yield. The final concentration was determined photometrically and the samples were stored at −70°C.

### cDNA synthesis, RT-PCR and sequence analysis

Equal amounts of total RNA of different stages were reverse transcribed with the *SuperScript™ II First-Strand Synthesis System* (Invitrogen) according to the manufacturer's instructions using random hexamer primers. For each RNA sample, one reaction was prepared which included *SuperScript™ II reverse transcriptase* (+RT), and one in which the enzyme was omitted (−RT). The cDNA samples were stored at −20°C until further analysis.

cDNA was amplified with the degenerate primer pairs RIFAFor/RIFARev and RIFBFor/RIFBRev as previously described [24] or with specific primer pairs PFD1240wVFor (TGGACAAATTATGTTACACAAACG)/ PFD1240wVRev (AGTATTAGTTAATATA GTAGTTTTTGG) and PFI0025cVFor (AATATCTGGAAACCTACGG)/ PFI0025cVRev (AGCAGTCTACAAATAGCAT). Genomic DNA (gDNA) was amplified in parallel as a control. Both, cDNA and gDNA products generated with degenerate primers were purified from agarose gels and cloned into the pCR2.1 TOPO TA vector (Invitrogen). Single colonies were analyzed by sequencing and inserts identified using BLAST searches.

### Phylogenetic analysis

Nucleotide sequences of 5′-upstream regions as well as location and transcriptional directions of 3D7 *rif* genes were obtained from the *Plasmodium* data base at http://www.plasmodb.org. Alignments were performed using ClustalW [66] and corrected manually using the *Bioedit* software [67]. Phylogenetic reconstructions were performed with the p-distance neighbour-joining (NJ) as well as the maximum parsimony (MP) algorithms using the *MEGA 3.1* software package [68]. Gaps were treated as pairwise deletions. Trees were bootstrapped 500 times and compared between NJ and MP tree-building methods to assure confidence in topology.

Nucleotide sequences of 5′-upstream regions of *rif* genes from the HB3 genome were retrieved from the *P. falciparum* genome data base at the Broad Institute (http://www.broad. mit.edu/annotation/genome/plasmodium_falciparum_spp/Downloads.html).

### Calculation of relative *rups* group related transcript abundance

To calculate the relative abundance of transcripts representing each of the *rups* groups, the percentage of cloned *rups* related sequences was determined for all stages. The median of the three experiments was calculated and, in order to correct for variant frequencies in the genome, divided by the percentage of *rups* group related variants in the genome.

## Supporting Information

Table S1 Overview over rups groups and characteristics of associated *rif* genes.
(0.43 MB DOC)

Figure S1 Neighbour Joining Distance tree of 3D7 and HB3 *rif* gene 5′-upstream sequences. 1000 bp sequences were analyzed. Gaps were treated as pairwise deletions. Bootsrap values at nodes differentiating the rups clusters are indicated. Purple: rupsA2, green: rupsA1, light blue: rupsAB, yellow: rupsB, dark blue: rupsC. Sequences labelled with a star are A-type *rif* sequences clustering with rupsB.
(5.32 MB TIF)
