# Supplementary material for: Diverse Expression Patterns of Subgroups of the rif Multigene Family during Plasmodium falciparum Gametocytogenesis
Source: PLoS One. 2008 Nov 20;3(11):e3779. doi: 10.1371/journal.pone.0003779 (PMC2582490; doi:10.1371/journal.pone.0003779)
Supplement: Table S1 — Overview over rups groups and characteristics of associated rif genes. (0.43 MB DOC) [file pone.0003779.s001.doc]

Table S1: Overview over rups groups and characteristics of associated *rif* genes

| **PlasmoDB**  **ID** | **orienta-tion^1^** | **chrom. postition** | **rups NJ^2^** | **A / B** | **SP^3^** | **TM^4^** | **upstream**  **gene^5^** | **downstream**  **gene^5^** | **Trans-cription^6^** | **Mass Spec^6^** |
| --- | --- | --- | --- | --- | --- | --- | --- | --- | --- | --- |
| >PF10_0397 | tel | right tel 10 | rups A/B | **B** | yes | 1 | *rif* | *rif* | S,M,Sp |  |
| >PF10_0401 | tel | right tel 10 | rups A/B | **B** | yes | 1 | *rif* | *rif* | Sp | Sp |
| >PF10_0402 | tel | right tel 10 | rups A/B | **B** | yes | 1 | *rif* | *rif* |  |  |
| >PF14_0005 | tel | left tel 14 | rups A/B | **B** | yes | 1 | *rif* | *rif* | Sp |  |
| >PFI0050c | tel | left tel 9 | rups A/B | **B** | yes | 2 | *rif* | *stevor* |  | G |
| >PFL2630w | tel | right tel 12 | rups A/B | **B** | no | 1 | *rif* | *stevor* |  |  |
| >PFL2640c | cen | right tel 12 | rups A/B | **A** | no | 1 | *rif* | *stevor <->* |  |  |
| >MAL13P1.500 | tel | right tel 13 | rups A/B | **A** | no | 1 | *rif* | *stevor* |  |  |
| >PF08_0138 | tel | left tel 8 | rups A/B | **A** | yes | 1 | *hyp* | *rif* | Sp |  |
| >PF10_0398 | tel | right tel 10 | rups A/B | **A** | no | 1 | *rif* | *rif* | Sp |  |
| >PF10_0400 | tel | right tel 10 | rups A/B | **A** | no | 1 | *rif* | *rif* | Sp |  |
| >PF10_0403 | tel | right tel 10 | rups A/B | **A** | yes | 1 | *rif* | *rif* | Sp |  |
| >PF11_0021 | tel | left tel 11 | rups A/B | **A** | yes | 2 | *rif psi* | *rif* | Sp |  |
| >PF14_0004 | tel | left tel 14 | rups A/B | **A** | yes | 2 | *rif* | *rif* | Sp | Sp |
| >PFA0045c | tel | left tel 1 | rups A/B | **A** | yes | 1 | *rif* | *rif <->* |  |  |
| >PFA0740w | tel | right tel 1 | rups A/B | **A** | no | 1 | *hyp* | *rif* |  | Sp,G |
| >PFB1010w | tel | right tel 2 | rups A/B | **A** | no | 1 | *rif* | *rif* |  |  |
| >PFD0040c | tel | left tel 4 | rups A/B | **A** | no | 1 | *rif* | *stevor* | T,Sp |  |
| >PFE0020c | tel | right tel 5 | rups A/B | **A** | no | 1 | *rif* | *rif psi* | Sp |  |
|  |  |  |  |  |  |  |  |  |  |  |
| >MAL13P1.520 | cen | right tel 13 | rups A1 | **A** | no | 1 | *hyp <->* | *rif <->* |  |  |
| >MAL7P1.216 | cen | left tel 7 | rups A1^7^ | **A** | yes | 1 | *rif <->* | *rif <->* |  |  |
| >PF11_0009 | cen | left tel 11 | rups A1^7^ | **A** | no | 1 | *var <->* | *rif* |  |  |
| >PF11_0520 | cen | right tel 11 | rups A1^7^ | **A** | no | 1 | *var <->* | *rif* |  |  |
| >PF13_0004 | cen | left tel 13 | rups A1^7^ | **A** | no | 1 | *var <->* | *rif* |  |  |
| >PFA0020w | cen | left tel 1 | rups A1^7^ | **A** | no | 1 | *var like <->* | *var psi <->* | T,G |  |
| >PFD0025w | cen | left tel 4 | rups A1^7^ | **A** | no | 1 | *var <->* | *rif <->* |  |  |
| >PFD1230c | cen | right tel 4 | rups A1^7^ | **A** | no | 1 | *var <->* | *rif psi <->* |  | G |
| >PFF0025w | cen | left tel 6 | rups A1^7^ | **A** | yes | 1 | *var <->* | *var psi <->* |  |  |
| >PFF1565c | cen | right tel 6 | rups A1 | **A** | no | 2 | *rif <->* | *rif* |  |  |
| >PFI0065w | cen | left tel 9 | rups A1 | **A** | no | 1 | *hyp <->* | *rif* | Sp |  |
| >PFL2645c | cen | right tel 12 | rups A1 | **A** | no | 1 | *hyp <->* | *rif* |  |  |
| >MAL7P1.184 | cen | right tel 7 | rups A1 | **A** | no | 1 | *rif* | *rif <->* |  |  |
| >MAL7P1.185 | cen | right tel 7 | rups A1 | **A** | yes | 1 | *hyp <->* | *rif* |  |  |
| >PF11_0519 | cen | right tel 11 | rups A1 | **A** | no | 1 | *rif* | *rif psi <->* |  |  |
| >PFA0040w | cen | left tel 1 | rups A1 | **A** | no | 1 | *hyp <->* | *rif <->* |  |  |
| >PFB0060w | cen | left tel 2 | rups A1 | **A** | yes | 1 | *hyp <->* | *stevor* |  |  |
| >PFC0035w | cen | left tel 3 | rups A1 | **A** | yes | 1 | *rif <->* | *rif* |  | Sp |
| >PFD0050w | cen | left tel 4 | rups A1 | **A** | no | 1 | *rif <->* | *rif* |  | Sp |
| >PFI0075w | cen | left tel 9 | rups A1 | **A** | yes | 1 | *rif* | *stevor* | Sp |  |
|  |  |  |  |  |  |  |  |  |  |  |
| >PFD0640c | tel left | central 4 | rups C | **A** | yes | 1 | *rif <->* | *var* |  |  |
| >PFF0855c | tel left | central 6 | rups C | **A** | no | 1 | *hyp <->* | *stevor* |  | Sp |
| >PFD0645w | tel right | central 4 | rups C | **A** | yes | 1 | *rif <->* | *hyp* |  |  |
| >PFD1020c | cen | central 4 | rups C | **A** | yes | 0 | *var <->* |  | Sp |  |
| >MAL7P1.57 | cen | central 7 | rups C | **A** | yes | 1 | *var <->* | *hyp* |  |  |
|  |  |  |  |  |  |  |  |  |  |  |
| >MAL13P1.515 | tel | right tel 13 | rups A2 | **A** | no | 1 | *var psi* | *rif <->* |  |  |
| >PF07_0132 | tel | right tel 7 | rups A2 | **A** | yes | 2 | *var psi* | *rif <->* |  |  |
| **PlasmoDB**  **ID** | **orienta-tion^1^** | **chrom. postition** | **rups NJ^2^** | **A / B** | **SP^3^** | **TM^4^** | **upstream**  **gene^5^** | **downstream**  **gene^5^** | **Trans-cription^6^** | **Mass Spec^6^** |
| >PFA0760w | tel | right tel 1 | rups A2 | **A** | no | 1 | *var psi* | *var <->* | R,T,Sp |  |
| >PF14_0769 | tel | right tel 14 | rups A2 | **A** | no | 1 | *rif* | *rif* |  |  |
| >PFB0030c | tel | left tel 2 | rups A2 | **A** | no | 1 | *rif* | *stevor* | Sp |  |
| >PFB0040c | tel | left tel 2 | rups A2 | **A** | no | 1 | *var psi* | *rif* | Sp | Sp |
| >PFB1035w | tel | right tel 2 | rups A2 | **A** | no | 1 | *hyp* | *var* |  |  |
| >MAL13P1.2 | tel | left tel 13 | rups A2 | **A** | no | 1 | *var* | *var <->* |  |  |
| >MAL13P1.535 | tel | right tel 13 | rups A2 | **A** | no | 1 | *rif* | *var <->* |  |  |
| >MAL7P1.213 | tel | left tel 7 | rups A2 | **A** | no | 1 | *var psi* | *var <->* |  |  |
| >MAL8P1.208 | tel | right tel 8 | rups A2 | **A** | no | 1 | *var* | *var psi <->* |  |  |
| >PF07_0138 | tel | right tel 7 | rups A2 | **A** | no | 1 | *var psi* | *var <->* |  |  |
| >PF11_0529 | tel | left tel 11 | rups A2 | **A** | no | 1 | *rif* | *var psi <->* | T |  |
| >PFA0010c | tel | left tel 1 | rups A2 | **A** | no | 1 | *var like* | *var <->* | T,Sp |  |
| >PFB1050w | tel | right tel 2 | rups A2 | **A** | no | 1 | *var psi* | *var <->* | T,Sp | Sp |
| >PFC0010c | tel | left tel 3 | rups A2 | **A** | no | 1 | *var psi* | *var <->* |  |  |
| >PFD0015c | tel | left tel 4 | rups A2 | **A** | no | 1 | *var* | *var <->* |  | Sp |
| >PFD0030c | tel | left tel 4 | rups A2 | **A** | no | 1 | *stevor* | *rif <->* | Sp |  |
| >PFD1240w | tel | right tel 4 | rups A2 | **A** | no | 1 | *var* | *var <->* | T,S |  |
| >PFF0015c | tel | left tel 6 | rups A2 | **A** | no | 1 | *var* | *var <->* |  |  |
| >PFF0035c | tel | left tel 6 | rups A2 | **A** | no | 1 | *rif psi* | *var psi <->* |  |  |
| >PFF1590w | tel | right tel 6 | rups A2 | **A** | no | 1 | *rif psi* | *var <->* |  |  |
| >PFI0010c | tel | left tel 9 | rups A2 | **A** | no | 1 | *rif* | *var <->* |  | Sp |
| >PFI0030c | tel | left tel 9 | rups A2 | **A** | no | 1 | *rif* | *rif* |  |  |
| >PFI1825w | tel | right tel 9 | rups A2 | **A** | no | 1 | *var* | *var <->* |  |  |
| >PFL0010c | tel | left tel 12 | rups A2 | **A** | no | 1 | *rif* | *var <->* | Sp |  |
| >PFL2615w | tel | right tel 12 | rups A2 | **A** | no | 1 | *stevor* | *stevor* | Sp | Sp |
| >PFL2660w | tel | right tel 12 | rups A2 | **A** | no | 1 | *rif* | *var <->* | Sp |  |
| >MAL8P1.219 | cen | right tel 8 | rups A2 | **A** | no | 1 | *var* | *rif <->* |  |  |
| >PF11_0010 | cen | left tel 11 | rups A2 | **A** | no | 1 | *rif* | *rif <->* |  | Sp |
| >PF13_0005 | cen | left tel 13 | rups A2 | **A** | no | 1 | *rif* | *rif <->* |  |  |
| >PFC0040w | cen | left tel 3 | rups A2 | **A** | no | 1 | *rif* | *rif like* | Sp |  |
| >PFD0055w | cen | left tel 4 | rups A2 | **A** | no | 1 | *rif* | *rif* |  | Sp |
| >PFD0060w | cen | left tel 4 | rups A2 | **A** | yes | 1 | *rif* | *stevor psi* | Sp |  |
| >PFF1560c | cen | right tel 6 | rups A2 | **A** | no | 1 | *rif* | *rif <->* |  |  |
| >PFI0070w | cen | left tel 9 | rups A2 | **A** | yes | 1 | *rif* | *rif* |  |  |
| >PF07_0134 | cen | right tel 7 | rupsA2 | **A** | yes | 2 | *rif* | *rif* | T,Sp |  |
| >MAL7P1.217 | tel | left tel 7 | rups A2 | **A** | no | 1 | *stevor* | *rif <->* |  |  |
| >MAL8P1.218 | tel | right tel 8 | rups A2 | **A** | yes | 2 | *stevor* | *rif <->* |  |  |
| >PF10_0396 | tel | right tel 10 | rups A2 | **A** | yes | 1 | *stevor* | *rif* |  |  |
| >PF14_0006 | tel | left tel 14 | rups A2 | **A** | no | 1 | *stevor* | *rif* | T |  |
| >PF14_0772 | tel | right tel 14 | rups A2 | **A** | no | 1 | *stevor* | *var* |  |  |
| >PFF1575w | tel | right tel 6 | rups A2 | **A** | no | 1 | *rif* | *var <->* |  |  |
| >PFI0035c | tel | left tel 9 | rups A2 | **A** | yes | 1 | *var psi* | *rif* | T,S,G,Sp |  |
| >PFL0025c | tel | left tel 12 | rups A2 | **A** | no | 1 | *var* | *var <->* |  |  |
| >PFL2625w | tel | right tel 12 | rups A2 | **A** | yes | 1 | *stevor* | *rif* | T,S,Sp |  |
| >PF10_0004 | tel | left tel 10 | rups A2 | **A** | no | 1 | *rif* | *rif* |  |  |
| >PF14_0002 | tel | left tel 14 | rupsA2 | **A** | no | 1 | *rif* | *var psi* | T,Sp | Sp |
| >MAL13P1.4 | tel | left tel 13 | rups A2 | **A** | no | 1 | *var psi* | *rif* |  |  |
|  |  |  |  |  |  |  |  |  |  |  |
| >MAL13P1.530 | tel | right tel 13 | rups B | **B** | yes | 1 | *hyp* | *rif* |  |  |
| >MAL7P1.215 | tel | left tel 7 | rups B | **B** | yes | 1 | *rif <->* | *var psi* |  |  |
| >MAL7P1.219 | tel | left tel 7 | rups B | **B** | no | 1 | *hyp* | *stevor* |  |  |
| **PlasmoDB**  **ID** | **orienta-tion^1^** | **chrom. postition** | **rups NJ^2^** | **A / B** | **SP^3^** | **TM^4^** | **upstream**  **gene^5^** | **downstream**  **gene^5^** | **Trans-cription^6^** | **Mass Spec^6^** |
| >PF10_0006 | tel | left tel 10 | rups B | **B** | yes | 2 | *hyp* | *rif psi* | T |  |
| >PF07_0136 | tel | right tel 7 | rups B | **B** | yes | 2 | *hyp* | *var* |  | Sp |
| >PF10_0394 | tel | right tel 10 | rups B | **B** | yes | 1 | *rif* | *stevor* |  |  |
| >PF10_0399 | tel | right tel 10 | rups B | **B** | yes | 1 | *rif* | *rif* | R,T,S,Sp |  |
| >PF10_0404 | tel | right tel 10 | rups B | **B** | no | 1 | *rif* | *rif* | Sp |  |
| >PF11_0020 | tel | left tel 11 | rups B | **B** | yes | 1 | *rif* | *rif* | Sp |  |
| >PF11_0515 | tel | right tel 11 | rups B | **B** | yes | 1 | *hyp* | *stevor* | Sp |  |
| >PF13_0006 | tel | left tel 13 | rups B | **B** | yes | 1 | *rif <->* | *rif* | G,Sp | Sp |
| >PF14_0003 | tel | left tel 14 | rups B | **B** | yes | 2 | *rif* | *rif* | T,Sp | Sp |
| >PF14_0008 | tel | left tel 14 | rups B | **B** | yes | 2 | *stevor* | *hyp* |  |  |
| >PF14_0766 | tel | right tel 14 | rups B | **B** | yes | 2 | *hyp* | *stevor* | Sp |  |
| >PF14_0770 | tel | right tel 14 | rups B | **B** | yes | 1 | *rif* | *stevor* | Sp |  |
| >PFA0030c | tel | left tel 1 | rups B | **B** | yes | 1 | *hyp* | *var psi* |  |  |
| >PFA0095c | tel | left tel 1 | rups B | **B** | yes | 1 | *hyp* | *stevor* |  |  |
| >PFA0710c | cen | right tel 1 | rups B | **B** | yes | 1 | *hyp* | *stevor* |  | G,M |
| >PFA0745w | tel | right tel 1 | rups B | **B** | yes | 1 | *rif* | *stevor* | Sp |  |
| >PFB0055c | tel | left tel 2 | rups B | **B** | yes | 2 | *hyp* | *stevor* |  |  |
| >PFB1005w | tel | right tel 2 | rups B | **B** | yes | 1 | *rif* | *rif* |  |  |
| >PFC0030c | tel | left tel 3 | rups B | **B** | yes | 1 | *rif <->* | *stevor* |  |  |
| >PFC1100w | tel | right tel 3 | rups B | **B** | yes | 2 | *rif* | *stevor* |  |  |
| >PFD0045c | tel | left tel 4 | rups B | **B** | yes | 1 | *rif <->* | *rif* | M,Sp |  |
| >PFD1010w | tel | right tel 4 | rups B | **B** | yes | 1 | *var <->* | *var <->* | Sp |  |
| >PFE0025c | tel | left tel 5 | rups B | **B** | no | 2 | *stevor psi* | *rif* |  |  |
| >PFF1570w | tel | right tel 6 | rups B | **B** | yes | 1 | *rif <->* | *rif* |  | G |
| >PFI0015c | tel | left tel 9 | rups B | **B** | yes | 1 | *rif <->* | *rif* | Sp | Sp,G,M |
| >PFI0025c | tel | left tel 9 | rups B | **B** | yes | 1 | *rif* | *rif <->* | S,M.Sp | ~ |
| >PFI1810w | tel | right tel 9 | rups B | **B** | yes | 1 | *rif* | *rif <->* |  | Sp |
| >PFL0015c | tel | left tel 12 | rups B | **B** | yes | 1 | *var <->* | *rif* | S,Sp | Sp |
| >PFL2605w | tel | right tel 12 | rups B | **B** | yes | 1 | *hyp* | *stevor* | Sp |  |
| >PFL2655w | tel | right tel 12 | rups B | **B** | yes | 2 | *hyp* | *rif* | S,M,G,Sp |  |
| >PFF1545w | tel | right tel 6 | rups B | **B** | yes | 1 | *rif psi* | *stevor* |  |  |
| >PF11_0011 | tel | left tel 11 | rups B | **A** | yes | 2 | *hyp* | *rif <->* |  |  |
| >PFI0055c | tel | left tel 9 | rups B | **A** | no | 1 | *hyp* | *rif* |  | M |
| >PFI1805w | tel | right tel 9 | rups B | **A** | no | 2 | *hyp* | *rif* | Sp |  |
| >MAL7P1.222 | cen | left tel 7 | rups B | **A** | yes | 1 | *stevor psi* | *stevor* |  |  |
|  |  |  |  |  |  |  |  |  |  |  |
| >PF10_0393 | tel | right tel 10 | x | **A** | yes | 1 | *hyp* | *rif* |  |  |
| >PFA0050c | tel | left tel 1 | x | **A** | yes | 1 | *hyp* | *rif* |  |  |
| >PF08_0105 | cen | left central 8 | x | **A** | yes | 1 | *var* | *rif* |  |  |
| >PFE1630w | tel | right tel 5 | x | **B** | yes | 2 | *var psi <->* | *rif psi* |  |  |
| >PFL2585c | cen | right tel 12 | x | **A** | yes | 1 | *hyp <->* | *rif psi <->* |  |  |
| >PF08_0104 | cen | left central 8 | x | **A** | no | 2 | *rif* | *var* | T,S,Sp |  |
| >PF07_0003 | tel | left tel 7 | x | **A** | no | 1 | *hyp* | *hyp* |  |  |
| >PFD0070c | tel | left tel 4 | x | **A** | yes | 2 | *-* | *stevor psi <->* | R,T,S,M |  |
| >PFB0035c | tel | left tel 2 | x | **B** | no | 1 | *rif* | *rif* | Sp |  |

^1^ transcription orientation (tel/cen: towards telomere/centromere); ^2^ rups NJ determined by Neighbor-Joining distance tree; ^3^ SP: signal peptide predicted by SignalP; ^4^ TM: transmembrane domain predicted by TMHMM 2.0; ^5^ adjacent genes with orientation in the same or opposite (<->) direction, psi: pseudogene; ^6^ Expression in developmental stages based on transcriptional and mass spectrometry evidence (R: ring, T: trophozoite, S: schizonts, M: merozoite, Sp: sporozoite, G: gametocyte) (Florens et al. 2002; Le Roch et al. 2003); ^7^ referred to as upsA-*rif* by Lavstsen et al. 2003.
