# Supplementary figures and images for: Diverse Expression Patterns of Subgroups of the rif Multigene Family during Plasmodium falciparum Gametocytogenesis
Source: PLoS One. 2008 Nov 20;3(11):e3779. doi: 10.1371/journal.pone.0003779 (PMC2582490; doi:10.1371/journal.pone.0003779)

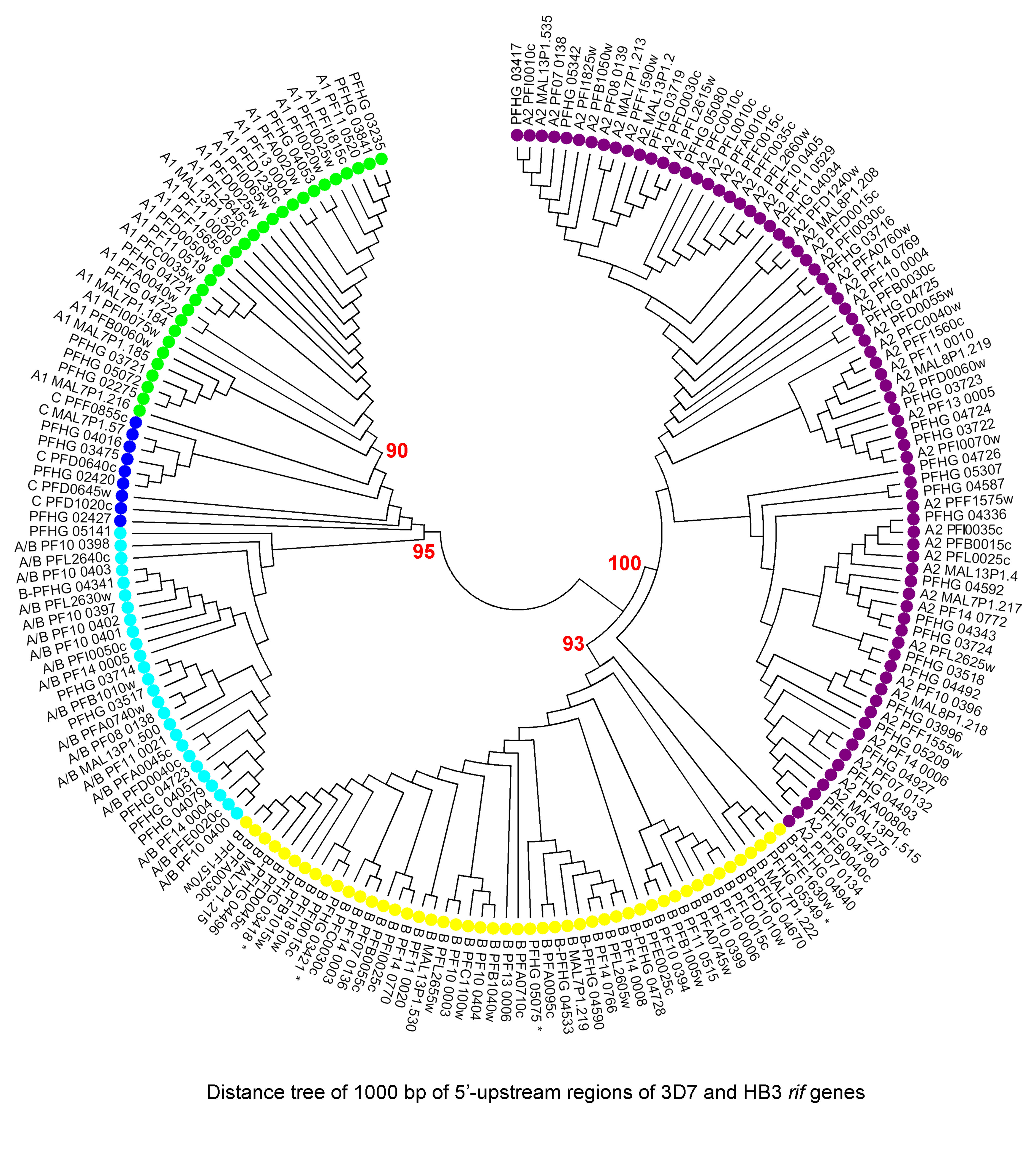

Supplement: Figure S1 — Neighbour Joining Distance tree of 3D7 and HB3 rif gene 5′-upstream sequences. 1000 bp sequences were analyzed. Gaps were treated as pairwise deletions. Bootsrap values at nodes differentiating the rups clusters are indicated. Purple: rupsA2, green: rupsA1, light blue: rupsAB, yellow: rupsB, dark blue: rupsC. Sequences labelled with a star are A-type rif sequences clustering with rupsB. (5.32 MB TIF) [file pone.0003779.s002.tif]
